# Lack of α_2C_-Adrenoceptor Results in Contrasting Phenotypes of Long Bones and Vertebra and Prevents the Thyrotoxicosis-Induced Osteopenia

**DOI:** 10.1371/journal.pone.0146795

**Published:** 2016-01-27

**Authors:** Marilia Bianca Cruz Grecco Teixeira, Gisele Miyamura Martins, Manuela Miranda-Rodrigues, Iasmin Ferreira De Araújo, Ricardo Oliveira, Patrícia Chakur Brum, Cecilia Helena Azevedo Gouveia

**Affiliations:** 1 Department of Anatomy, Institute of Biomedical Sciences, University of Sao Paulo, Sao Paulo, Brazil; 2 RDODiagnosticos Medicos, Sao Paulo, Brazil; 3 Departament of Biodinamic of Human Body Moviment, School of Physical Education and Sport, University of Sao Paulo, Sao Paulo, Brazil; Faculté de médecine de Nantes, FRANCE

## Abstract

A series of studies have demonstrated that activation of the sympathetic nervous system (SNS) causes osteopenia via β_2_-adrenoceptor (β_2_-AR) signaling. However, in a recent study, we found an unexpected and generalized phenotype of high bone mass in female mice with chronic sympathetic hyperactivity, due to double gene inactivation of adrenoceptors that negatively regulate norepinephrine release, α_2A_-and α_2C_-AR (α_2A/2C_-AR^-/-^). These findings suggest that β_2_-AR is not the single adrenoceptor involved in bone turnover regulation and show that α_2_-AR signaling may also mediate the SNS actions in the skeleton. In addition, we found that α_2A/2C_-AR^-/-^ animals are resistant to the thyrotoxicosis-induced osteopenia, suggesting that thyroid hormone (TH), when in supraphysiological levels, interacts with the SNS to control bone mass and structure, and that this interaction may also involve α_2_-AR signaling. In the present study, to further investigate these hypotheses and to discriminate the roles of α_2_-AR subtypes, we have evaluated the bone phenotype of mice with the single gene inactivation of α_2C_-AR subtype, which mRNA expression was previously shown to be down regulated by triiodothyronine (T3). A cohort of 30 day-old female α_2C_AR^-/-^ mice and their wild-type (WT) controls were treated with a supraphysiological dose of T3 for 30 or 90 days, which induced a thyrotoxic state in both mouse lineages. The micro-computed tomographic (μCT) analysis showed that α_2C_-AR^-/-^ mice present lower trabecular bone volume (BV/TV) and number (Tb.N), and increased trabecular separation (Tb.Sp) in the femur compared with WT mice; which was accompanied by decreased bone strength (determined by the three-point bending test) in the femur and tibia. The opposite was observed in the vertebra, where α_2C_-AR^-/-^ mice show increased BV/TV, Tb.N and trabecular thickness (Tb.Th), and decreased Tb.Sp, compared with WT animals. In spite of the contrasting bone phenotypes of the femur and L5, thyrotoxicosis negatively regulated most of the micro architectural features of the trabecular bone in both skeletal sites of WT, but not of α_2C_-AR^-/-^ mice. T3 treatment also decreased biomechanical properties (maximum load and ultimate load) in the femur and tibia of WT, but not of knockout mice. The mRNA expression of osteocalcin, a marker of mature osteoblasts, and tartrate-resistant acid phosphatase, which is expressed by osteoclasts and is involved in collagen degradation, was increased by T3 treatment only in WT, and not in α_2C_-AR^-/-^ mice. Altogether, these findings suggest that α_2C_-AR subtype mediates the effects of the SNS in the bone in a skeletal site-dependent manner, and that thyrotoxicosis depends on α_2C_-AR signaling to promote bone loss, which sustains the hypothesis of a TH-SNS interaction to modulate bone remodeling and structure.

## Introduction

It is well known that hyperthyroidism is one of the major causes of secondary osteoporosis [1,2]. Thyroid hormone (TH) stimulates both bone formation and resorption by regulating the activity of osteoblasts and osteoclasts, respectively. In hyperthyroidism, both bone formation and resorption are increased [3,4], but the latter predominates [5], favoring resorption [3], negative balance of calcium [6], and bone loss [7]. In contrast, in hypothyroidism, bone turnover is slowed and bone mass might be slightly increased [8,9]. The mechanisms through which TH regulates bone remodeling are not completely understood. It is known that TH can modulate bone metabolism indirectly, regulating the synthesis and/or secretion of other hormones and factors, such as growth hormone and IGF-I [10,11,12]. A body of evidence also suggests that TH acts directly in bone cells, modifying their proliferation and differentiation and/or modulating the expression of several bone-related genes [13,14,15]. It is generally accepted that most of T3 actions are mediated by its nuclear receptors, which were shown to be expressed in osteoblasts [16], osteoclasts [17], and chondrocytes [18].

Over the last 15 years, a series of studies has demonstrated that the sympathetic nervous system (SNS) also controls bone metabolism [19]. Evidence shows that the SNS activation negatively regulates bone formation and positively regulates bone resorption, via β_2_-AR signaling, leading to bone loss [20,21,22]. β_2_-AR mRNA expression was detected in osteoblastic and osteoclastic cells [20,23], and β_2_-AR knockout mice (β_2_-AR^-/-^), which do not present metabolic and endocrine abnormalities, present a generalized phenotype of high bone mass (HBM), with increased bone formation and decreased bone resorption [24]. In vitro studies, with bone cells derived from wild-type (WT) and β_2_-AR^-/-^ mice showed that the SNS limits bone formation by acting directly on osteoblasts and favors bone resorption by increasing expression in osteoblast progenitor cells of the osteoclast differentiation factor RANKL (receptor activator of nuclear factor kappa-B ligand) [24]. Accordingly, administration of propranolol, a β-blocker, and isoproterenol, a β-agonist, was demonstrated to increase and decrease bone mass, respectively, in adult animals [20,25,26].

On the other hand, later studies by our group have shown that female mice with chronic sympathetic hyperactivity, due to the double gene inactivation of adrenoceptors that negatively regulate norepinephrine release, α_2A_-AR and α_2C_-AR (α_2A_/α_2C_-AR^-/-^), present an unexpected phenotype of HBM, with decreased bone resorption and increased bone formation [27]. These findings suggest that β_2_-AR is not the single adrenoceptor involved in bone mass regulation and evoke that α_2A_-AR and/or α_2C_-AR signaling may also mediate the SNS actions in the skeleton.

There are three subtypes of α_2_ adrenoceptors, α_2A_-AR, α_2B_-AR and α_2C_-AR. All these receptors are expressed in the presynaptic membranes of adrenergic neurons, where they inhibit catecholamine release, acting, therefore, as autoreceptors [28]. They are also expressed in non-adrenergic neurons of the peripheral and central nervous system (CNS), where they can act as heteroceptors [28], inhibiting the release of many neurotransmitters, including serotonin [29], GABA [30] and dopamine [31]. All α_2_ receptor subtypes are also expressed in non-neuronal cells, where they have important roles, such as regulation of body temperature, intraocular pressure, lipolysis, insulin release, and pain perception [28,32,33,34]. All these receptors were also detected, by immunohistochemistry, in osteoblasts, osteocytes, osteoclasts and chondrocytes in histological sections of the femur and vertebra of mice. [27]. In addition, α_2A_-AR was recently detected in human osteoblasts and lining cells [35].

Considering that TH interacts with the SNS to control several physiological processes, including thermogenesis, lipolysis, and glycogenolysis [36]; and that treatment of hyperthyroid patients with propranolol corrects their hypercalcemia [37] and decreases their urinary excretion of hydroxyproline, a biochemical marker of bone resorption [38], we have raised the hypothesis that TH, when in supraphysiological levels, could also interact with the SNS to regulate bone remodeling. In fact, we have found that α_2A/2C_-AR^-/-^ animals are resistant to the thyrotoxicosis-induced osteopenia [39], which substantiates a TH-SNS interaction to control bone mass and suggests that this interaction depends on α_2A_-AR and/or α_2C_-AR signaling. To further investigate these hypotheses and to discriminated the roles of the different α_2_ isoforms, we have evaluated the bone phenotype of mice with the single gene inactivation of α_2C_ adrenoreceptor subtype (α_2C_-AR^-/-^), which mRNA expression was previously shown to be modulated by TH [39].

In the present study, we show that α_2C_-AR^-/-^ animals present critical skeletal alterations, with higher bone mass in the vertebra and lower bone mass in the femur, when compared with WT mice. In addition, these mice also showed to be resistant to the deleterious effects of thyrotoxicosis on bone microstructure and biomechanical properties, bringing new evidence that the regulation of bone remodeling by the SNS is extremely complex and that TH, when in supraphysiological levels, interacts with this system to control bone remodeling and structure in an α_2C_-AR subtype-dependent manner.

## Materials and Methods

### Animal maintenance and manipulation

A cohort of 30-day old female congenic α_2C_-AR knockout (KO) mice (α_2C_-AR^-/-^) in a C57BL6/J (B6) background and their WT controls were studied. The animals were considered young adults, since the pubertal maturation in B6 female mice begins when serum estradiol increases by day 26 after birth and is complete when vaginal opening occurs by day 31 [40]. The animals received an injection of T3 (Sigma-Aldrich, Germany), in a daily dose equivalent to 20 fold its physiological dose (20xT3 = 7g/ 100g body mass/day), for 30 or 90 days (n = 8–10 animals/group). T3 injections were administered intraperitoneally and at the same time each day. All animals were weighed once a week to monitor the changes in body mass over the experimental period and for adjusting the amount of hormone to be administered in order to maintain the supraphysiological dose of T3 (20xT3). All experimental procedures were performed in accordance with the guidelines of the Standing Committee on Animal Research of the University of São Paulo, which approved the study (Protocol n°. 35, page 85, book 02). At the end of the experimental period, the animals were euthanized, and the body length was measured from the tip of the snout to the base of the tail.

### Serum levels of thyroxine (T4) and T3

At the end of the experimental period, the animals were euthanized and the blood was collected. The serum was separated by centrifugation and immediately frozen. Total thyroxine (T4) and T3 serum levels were measured using radioimmunoassay commercial kits (RIA-gnost T4 and RIAgnost T3; CIS Bio International, Gif-sur-Yvette, France). For the T4 and T3 assays, standard curves were built in our laboratory with a pool of charcoal stripped mouse serum. The blood samples were always collected 2 hours after the last T3 administration.

### Heart mass

Immediately after the animals were euthanized, the heartwas dissected out and weighed for wet-mass determination. The heart samples were dehydrated at 60°C for48 hours and weighed again for dry-mass determination. All masses were expressed in milligrams per gram of body mass (BM).

### Micro-Computed Tomography (μCT) analysis

A bone sample representative of the axial and appendicular skeleton, the fifth lumbar vertebra (L5) and femur, respectively, were scanned using a μCT unit (SkyScan 1172, SkyScan, Aartselaar, Belgium), where they were rotated through 360° at a rotation step of 0.7 degree. The X-ray settings were standardized to 100 kV for the baseline vertebral body and distal femur specimens, with an exposure time of 590 ms.A 0.05-mm-thick aluminum filter and a beam-hardening algorithm were used to minimize beam-hardening artifacts. The bone parameters were obtained with CtAn Version 1.5 (SkyScan). Total trabecular area of L5 and the distal metaphysis of the femur were selected as the regions of interest (ROIs). The following 3D structural parameters of trabecular bone were determined: BV/TV (bone volume/tissue volume), Tb.Th (trabecular thickness), Tb.N (trabecular number), and TB.Sp (trabecular separation). The following morphometric variables of cortical bone were measured in 2D cross-sectional images of the vertebral body of L5 and in the diaphysis of the femur: T.Ar (Tissue area), B.Ar (Bone area), Medulary Area and Endosteal Perimeter.

### Three-point bending test

The right femurs and tibias were tested in the same orientation in an Instron testing machine (Model 3344, Instron Corporation, MA, USA). For the femurs, the anterior cortex was placed in compression and the posterior cortex in tension during the test; for the tibias, the lateral right cortex was placed in compression and the lateral left cortex in tension during the test. A constant displacement rate of 5 mm/minute was applied until the bone fractured. Fracture was taken as complete loss of load carrying ability. To stabilize the specimen, a small preload (5% of the average maximal load) was applied before actual testing. During the bending test, load-displacement data were collected by a computerized data-acquisition system at a sampling rate of 80 Hz. The biomechanical properties evaluated were the maximum load [a measure of the maximum force that the bone withstood before fracture (N)], the ultimate load [the load at the fracture point (J)], Young´s modulus (mPa), resilience [a measure of the ability of a bone to suffer elastic deformity (J)] and stiffness [a measure of the extrinsic rigidity of the bone tissue (N/mm)].

### Gene expression by Real Time PCR

Expression of receptor activator of nuclear factor kappa-B (RANK), RANK ligand (RANKL), osteoprotegerin (OPG), osteocalcin (OC) and tartrate-resistant acid phosphatase (TRAP) were determined by real time PCR in the whole femur and fourth lumbar vertebra (L4), as described previously [41]. The total RNA was extracted from these whole bones, including the bone marrow, using Trizol (Invitrogen, Carlsbad, CA, USA), following the manufacturer´s instructions. Total RNA was reverse transcribed using TURBO DNA-free kit (Ambion, Foster City, CA, USA). Syber Green Super Mix (Applied Biosystems, Warrington, UK) was used for the real-time PCR using the ABI Prism 7500 sequence detector. All primers used in this study: RANK_F: TCT GCA GCT CTT CCA TGA CAC T and R: CGA TGA GAC TGG GCA GGT AAG (NM_009399), RANK-L_F: GGC CAC AGC GCT TCT CAG and R: GAG TGA CTT TAT GGG AAC CCG AT (NM_011613.2), OPG_F: AGT CCG TGA AGC AGG AGT G and R: CCA TCT GGA CAT TTT TTG CAA A (NM_U94331), 18S_F: GTA ACC CGT TGA ACC CCA TT and R: CCA TCC AAT CGG TAG TAG CG (NM_11188), TRAP_F: *F*: TGC ACA GAT TGC ATA CTC TAA GAT CTC, *R*: TTT TGA AGC GCA AAC GGT AGT (NM_007388), OC_F: *F*: CTC ACA GAT GCC AAG CCC A, *R*: CCA AGG TAG CGC CGG AGT CT (NM_U11542) were synthetized (Integrated DNA Technologies, Coralville, IA) specifically for real-time PCR using Primer Express software (Applied Biosystems). The amplification reaction was carried out for 40 cycles, with denaturation at 95°C for 5 seconds, and annealing/extension at 60°C for 31 seconds. Melt curve analysis was conducted after each run. Each pair of primer generated a single peak. The relative abundance of each target was calculated as 1,000 × 2(Ct target gene–Ct Gapdh), in which *C*_t_ represents the threshold cycle for each transcript, and Cyclophilin or 18s is the reference.

## Results

### Induction of a thyrotoxic state in α_2C_-AR^-/-^ mice

**Fig 1:**
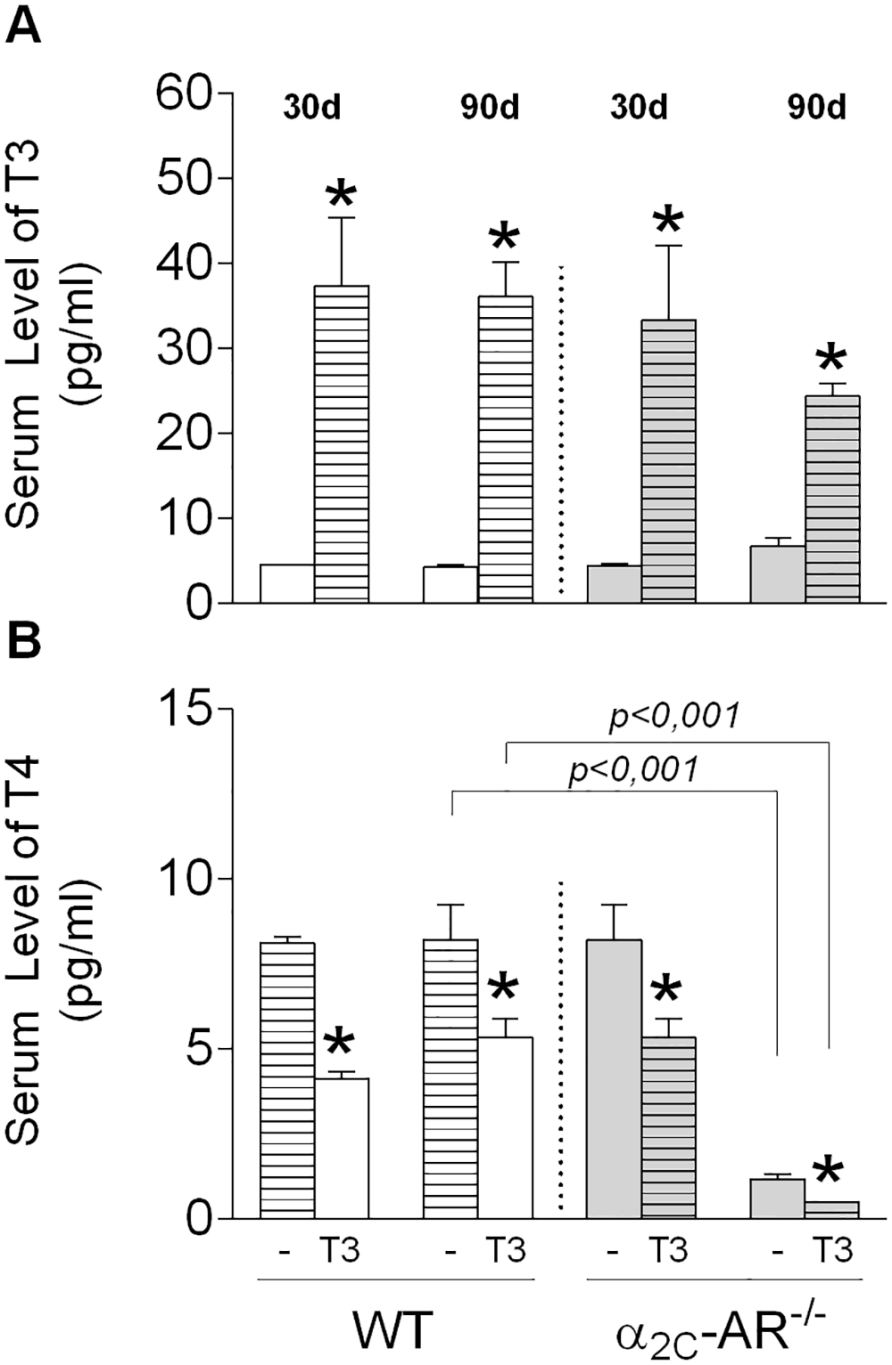
T3 and T4 serum levels in WT and α_2C_-AR^-/-^ mice. (A) Serum levels of T3. (B) Serum levels of T4. Animals were treated with saline or a supraphysiological dose of T3 (7 μg·100 g body wt^-1^·day^-1^). Significance between groups was determined by two-way ANOVA followed by Tukey’s test. Values are expressed as means ± SEM (*n* = 8-10/group). *P <0.05, *versus* the respective saline-treated animals (WT *vs*. WT+T3, KO *vs*. KO+T3).

**Fig 2:**
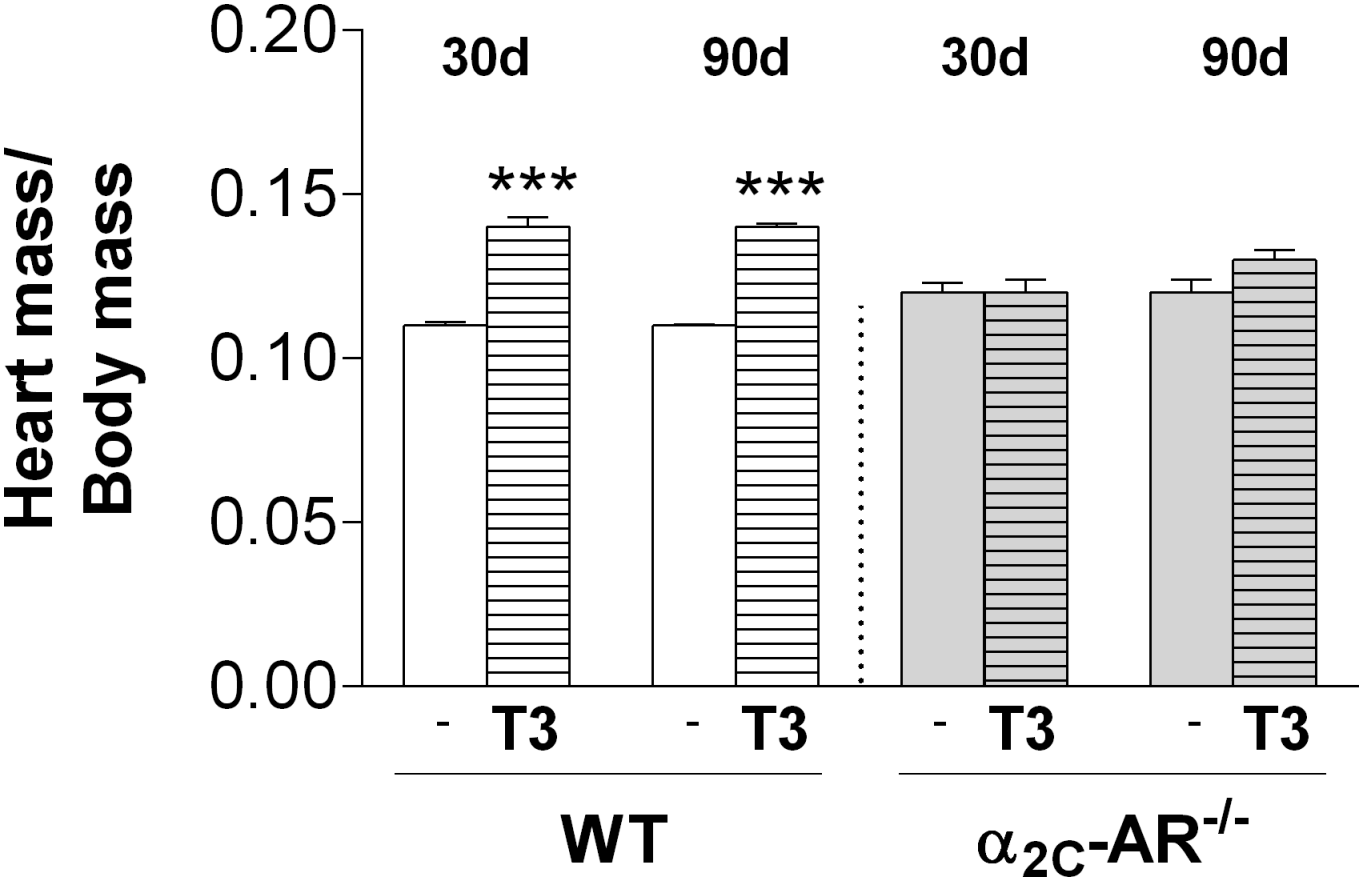
Effect of T3-treatment on heart mass of WT and α_2C_-AR^-/-^ mice. 30d and 90d, refer, respectively, to 30 and 90 days of treatment with saline or a supraphysiological dose of T3 (7 μg·100 g body wt^-1^·day^-1^). The dependent values were calculated dividing heart mass per body mass [(g of heart mass/g of body mass) x 100]. Significance between groups was determined by two-way ANOVA followed by Tukey’s test. Values are expressed as means ± SEM (*n* = 10–12 per group). ***P <0.001 *versus* the respective saline-treated animals (WT *vs*. WT+T3, KO *vs*. KO+T3).

**Fig 3:**
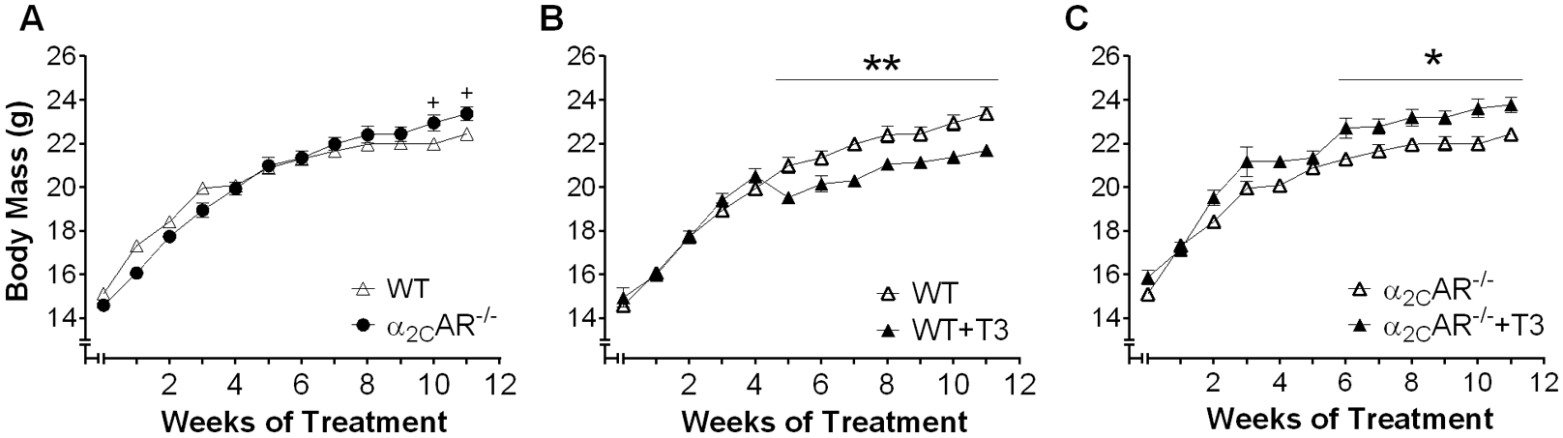
Body mass of WT and α_2C_-AR^-/-^ mice. (A) WT *versus* α_2C_-AR^-/-^ mice. (B) WT *versus* WT+T3. (C) α_2C_-AR^-/-^*versus* α_2C_-AR^-/-^+T3. Animals were treated with saline or a supraphysiological dose of T3 (7 μg·100 g body wt^-1^·day^-1^). The significance between all groups, presented in figures A, B and C, was determined in a single test by two-way ANOVA, followed by Tukey’s test. The groups were separated in figures A, B and C to more clearly show the differences between groups. Values represent the mean ± SEM (n = 10–12 per group). *P< 0.05, **P< 0.01, *versus* the respective saline-treated animals (WT *vs*. WT+T3, KO *vs*. KO+T3), ^+^*P* < 0.05 (WT vs. KO).

We evaluate the effect of daily administration of 20 times the physiological dose of T3 (20 X T3 = 7 μg x 100 g bw^-1^ x day^-1^) on bone of 30-day old female WT and α_2C_-AR^-/-^ mice for 30 and 90 days. We first evaluated the induction of a thyrotoxic state by analyzing serum levels of TH, heart mass and body mass. Both 30- and 90-day-long T3 treatments increased by 8-fold the serum concentration of T3, and, as expected, decreased by half the T4 serum levels both in WT and KO mice (Fig 1A and 1B), which reflects TSH inhibition by negative feedback [42]. Serum levels of T4 were not different between WT and KO mice (receiving saline or T3) by 60 days of age (30-day treatment group). On the other hand, by 120 days of age (90-day treatment group), saline- and T3-treated α_2C_-AR^-/-^ animals (Fig 1B) presented important decrease on T4 levels (7- fold and 9-fold, respectively), compared with their WT controls (saline–and T3-treated mice), suggesting that the lack of α_2C_-AR leads to a decline in the thyroid function, as the animals age. Corroborating previous studies that report cardiac hypertrophy in conditions of thyrotoxicosis [43], the 30-day and 90-day T3 treatments significantly increased heart mass in WT mice by 20% (*p*<0,001), which is an indirect measure of cardiac hypertrophy. Surprisingly, the heart mass of KO mice was not affected by the thyrotoxic state (Fig 2). Body mass was quite similar between saline-treated WT and KO mice (Fig 3A), but an unexpected result was also observed regarding to the effect of T3 on this parameter. As predicted, body mass decreased by 10–15%, as a consequence of the thyrotoxic state in WT mice (Fig 3B), which was clearly observed during the last 7 weeks of T3 treatment. In contrast, T3-treated α_2C_-AR^-/-^ mice showed a 6–9% increase in body mass compared with untreated KO mice (Fig 3C). These latter results also suggest a participation of α_2C_-AR in the thyrotoxicosis-induced cardiac hypertrophy and body mass loss, two well known consequences of a chronic thyrotoxic condition [43,44].

### T3 effects on bone microarchitecture of WT and α_2C_-AR^-/-^ mice

**Fig 4:**
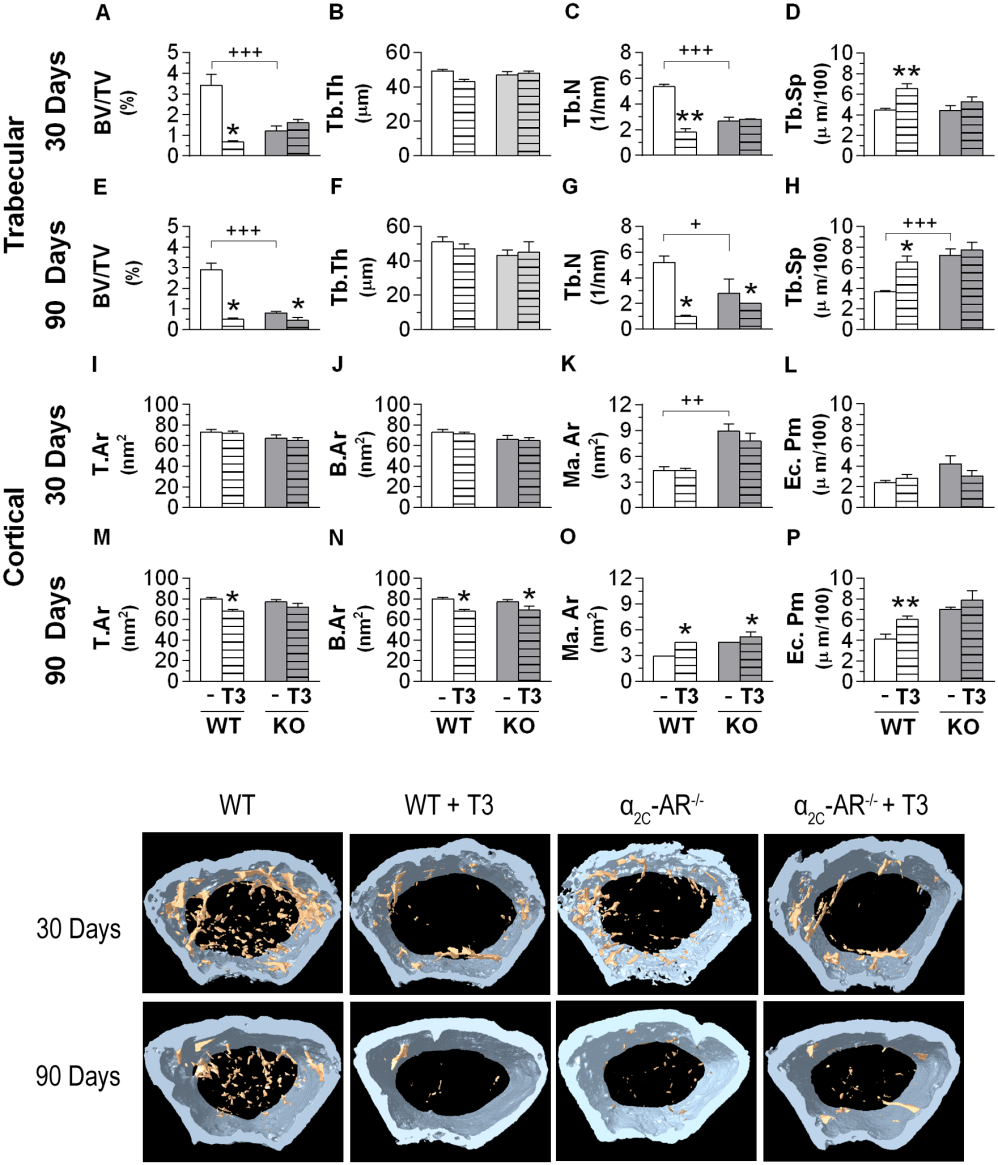
Effect of T3-treatment on the structural parameters of the trabecular and cortical bone of the distal metaphysis of the femur in WT and α_2C_-AR^-/-^ mice assessed by μCT. (A–D) Effect of 30-day treatment on trabecular parameters. (E-H) Effect of 90-day treatment on trabecular parameters. (I-L) Effect of 30-day treatment on cortical parameters. (M-P) Effect of 90-day treatment on cortical parameters. Animals were treated with saline or a supraphysiological dose of T3 (7 μg·100 g body wt^-1^·day^-1^). Significance between groups was determined by two-way ANOVA followed by Tukey’s test. Values are expressed as means ± SEM (*n* = 10–12 per group). *P<0.05 and **P< 0.01 *vs*. the respective saline-treated animals (WT *vs*. WT+T3, KO *vs*. KO+T3). ^+^*P*< 0.05, ^++^*P*< 0.01 and ^+++^*P*< 0.001 for differences between WT and KO mice, as indicated. BV/TV, trabecular bone volume; Tb.Th, trabecular thickness; Tb.N, trabecular number; Tb.Sp, trabecular speculation; T.Ar, total area; B.Ar, bone area; Ma.Ar, medullary area; Ec.Pm, endocortical perimeter.

**Fig 5:**
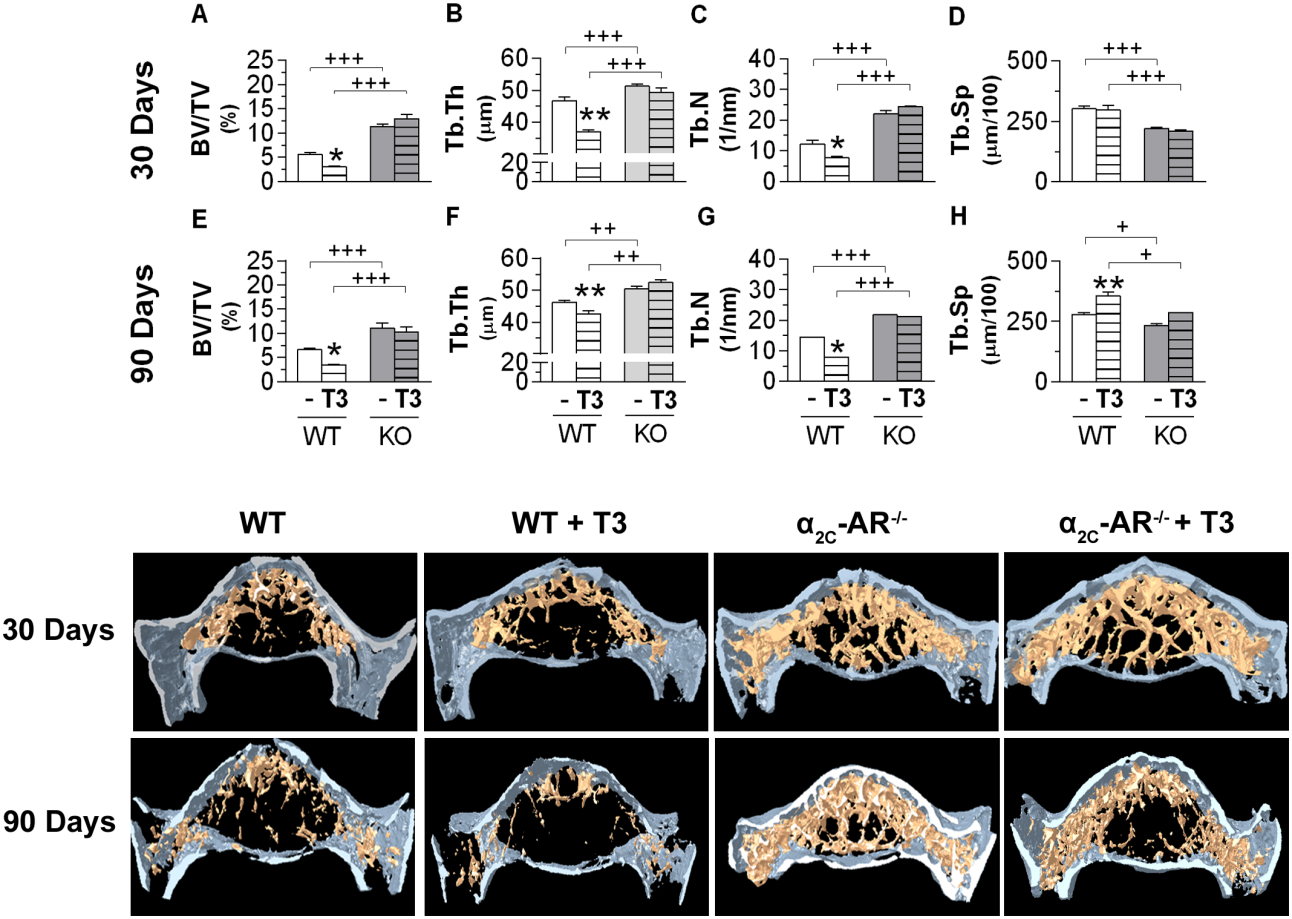
Effect of T3-treatment on the structural parameters of the trabecular bone of the vertebral body of L5 in WT and α_2C_-AR^-/-^ mice assessed by μCT. (A–D) Effect of 30-day treatment on trabecular parameters. (E-H) Effect of 90-day treatment on trabecular parameters. (I-L) Effect of 30-day treatment on cortical parameters. (M-P) Effect of 90-day treatment on cortical parameters. Animals were treated with saline or a supraphysiological dose of T3 (7 μg·100 g body wt^-1^·day^-1^). Significance between groups was determined by two-way ANOVA followed by Tukey’s test. Values are expressed as means ± SEM (*n* = 10–12 per group). *P <0.05 and **P< 0.01 *vs*. the respective saline-treated animals (WT *vs*. WT+T3, KO *vs*. KO+T3). ^+^*P*< 0.05, ^++^*P*< 0.01 and ^+++^*P*< 0.001 for differences between WT and KO mice, as indicated. BV/TV, trabecular bone volume; Tb.Th, trabecular thickness; Tb.N, trabecular number; Tb.Sp, trabecular speculation.

Significant differences in trabecular bone microarchitecture were observed between WT and KO mice, whereas cortical bone showed to be quite similar between these two mice strains (Figs 4–5 and S1 Fig). The μCT analysis of the distal methaphysis of the femur showed that the 30-day and 90-day saline-treated α_2C_-AR^-/-^ mice (60-day and 120-day old animals, respectively) presented lower BV/TV (1.8-fold and 8-fold, respectively) and Tb.N (70% and 85%, respectively) than their WT controls (Fig 4A, 4E, 4C and 4G), whereas Tb.Sp was increased by 95% in the 90-day saline-treated KO mice (Fig 4H). In contrast, the analysis of the lumbar vertebra (L5) showed the opposite (Fig 5A–5H). The 30-day and 90-day saline-treated KO mice presented higher BV/TV (55 and 48%, respectively), Tb.Th (10 and 9%, respectively) and Tb.N (80 and 40%, respectively); and lower Tb.Sp (27 and 16%, respectively) than their WT controls. In spite of these site skeleton differences (low and high trabecular bone mass in the femur and vertebra of KO mice, respectively, compared with WT mice), α_2C_-AR^-/-^ mice showed to be resistant to the deleterious effects of thyrotoxicosis on the trabecular bone of both femur and vertebra (Figs 4A–4H and 5A–5H). In contrast and, as expected, in WT animals, T3 treatment negatively affected the trabecular μCT parameters in both skeletal sites, but mainly in the femur, which is known to be more sensitive to the toxic effects of T3 [45,46]. 30- and 90-day T3-treament decreased femoral BV/TV (5-fold and 6-fold, respectively) and Tb,N (3-fold and 5-fold, respectively), and increased femoral Tb.Sp (47 and 80%, respectively) in WT animals (Fig 4A, 4C, 4D, 4E, 4G and 4H). In the vertebra of WT animals (Fig 5), 30- and 90-day T3-treament decreased BV/TV (60 and 50%, respectively), Tb.Th (30 and 10%, respectively) and Tb.N (43 and 49%, respectively), and increased Tb.Sp (27%, only after 90 days of T3-treatment). As commented above, all these negative effects of T3 were not observed in the vertebra of α_2C_-AR^-/-^ mice or were poorly observed in the femur of KO mice: only BV/TV and Tb.N were decreased (2 and 1.4 times, respectively) by T3 treatment in the femur KO animals, it occurred only after 90 days of treatment (whereas decreases in these parameters occurred after 30 and 90 days of treatment in WT animals) and in a much lower magnitude than in WT animals. Although less evident, the cortical bone was also differentially affected, in some way, by TH excess in the femur and vertebra of KO animals. In the femur, T.Ar decreased 16% and Ec.Pm increased 25% only in WT animals. All the other cortical parameters responded similarly to thyrotoxicosis in WT and KO animals (Fig 4I–4P). The thyrotoxic status also decreased some cortical parameters of the vertebra of WT mice, such as T.Ar (8–36%) and B.Ar (24–35%). Once again, KO mice showed to be resistant to these T3 effects (S1 Fig).

### TH effects on the bone biomechanical parameters of WT and α_2C_-AR^-/-^ mice

**Fig 6:**
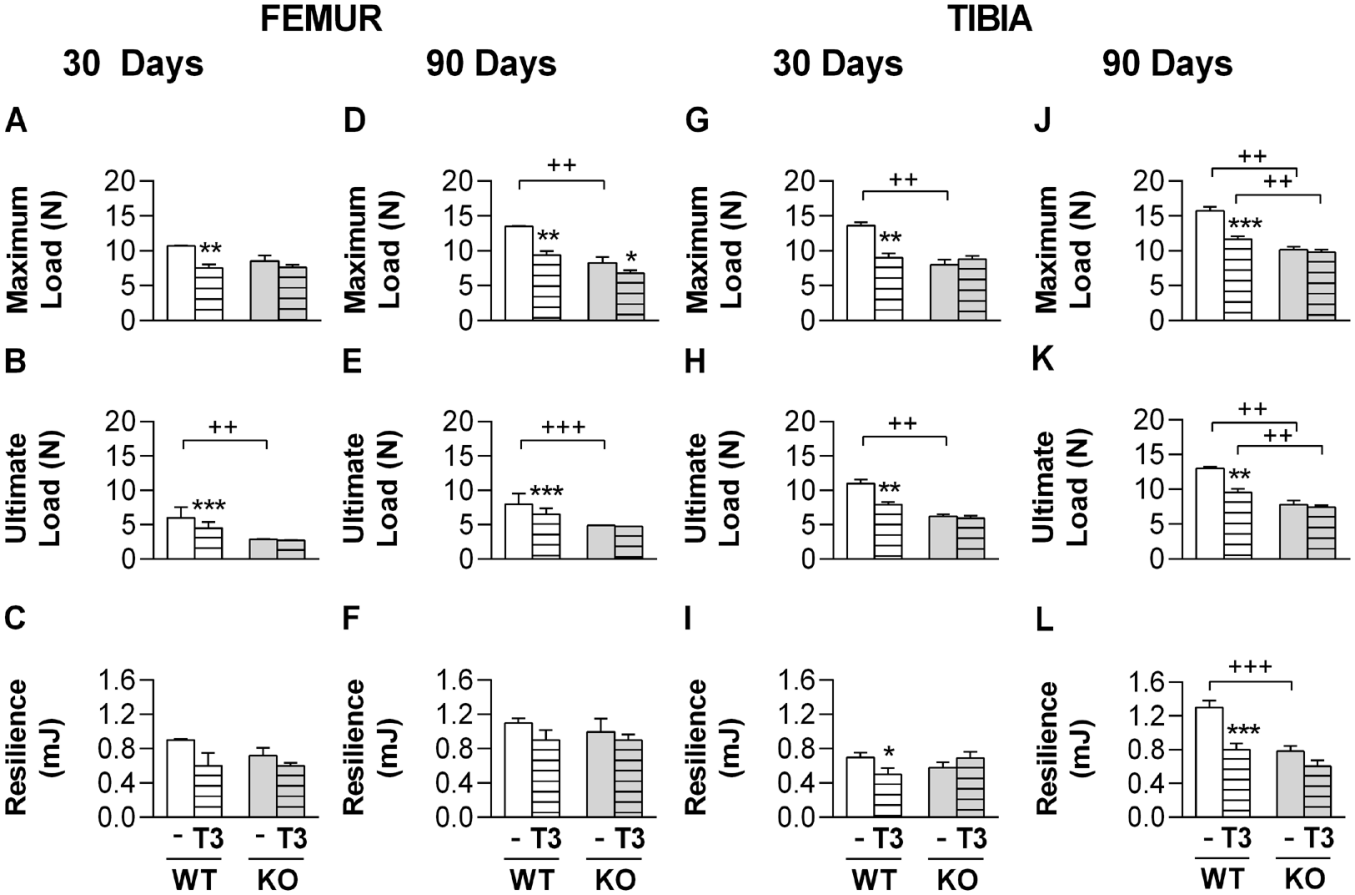
Effect of T3-treatment on the biomechanical parameters of the femur and tibia in WT mice and α_2C_-AR^-/-^ mice. Data were assessed by means of the 3-point bending test. Animals were treated with saline or a supraphysiological dose of T3 (7 μg·100 g body wt^-1^·day^-1^). Values are expressed as means ± SEM (*n* = 10–12 per group). Significance between groups was determined by two-way ANOVA followed by Tukey’s test. *P <0.05 and **P< 0.01 vs. the respective saline-treated animals (WT *vs*. WT+T3, KO *vs*. KO+T3). ^+^*P*< 0.05, ^++^*P* < 0.01 and ^+++^*P* < 0.001 for differences between WT and KO mice, as indicated.

In agreement with the μCT analysis that revealed lower BV/TV and Tb.N in the femur of α_2C_-AR^-/-^ mice, the three-point bending test showed that maximum load and ultimate load, which are measures of bone strength, were significantly lower in the femur and tibia of KO mice, as compared with WT controls (Fig 6B, 6D, 6E, 6G, 6H, 6J and 6K). Resilience was significantly lower only in the tibia of 120-old KO mice as compared with WT mice (Fig 6L). Once again corroborating the μCT findings, 30 or 90 days of T3 treatment significantly decreased femoral and tibial bone strength (maximum load and ultimate load) in WT animals, but not in KO mice (Fig 6A, 6B, 6E, 6G, 6H, 6J and 6K), except for femoral maximum load that was also decreased by 90 days of T3-treatment in KO animals (Fig 6D). Resilience, which reflects the elasticity of a material, was decreased by T3 treatment (30- and 90-day long) in the tibia of WT mice, but not in the tibia or femur of α_2C_-AR^-/-^mice (Fig 6I and 6L). Altogether, these data reinforce the bone resistance of KO mice to the negative effects of TH excess.

### TH effects on the gene expression of the RANKL-RANK-OPG system and on bone remodeling markers

**Fig 7:**
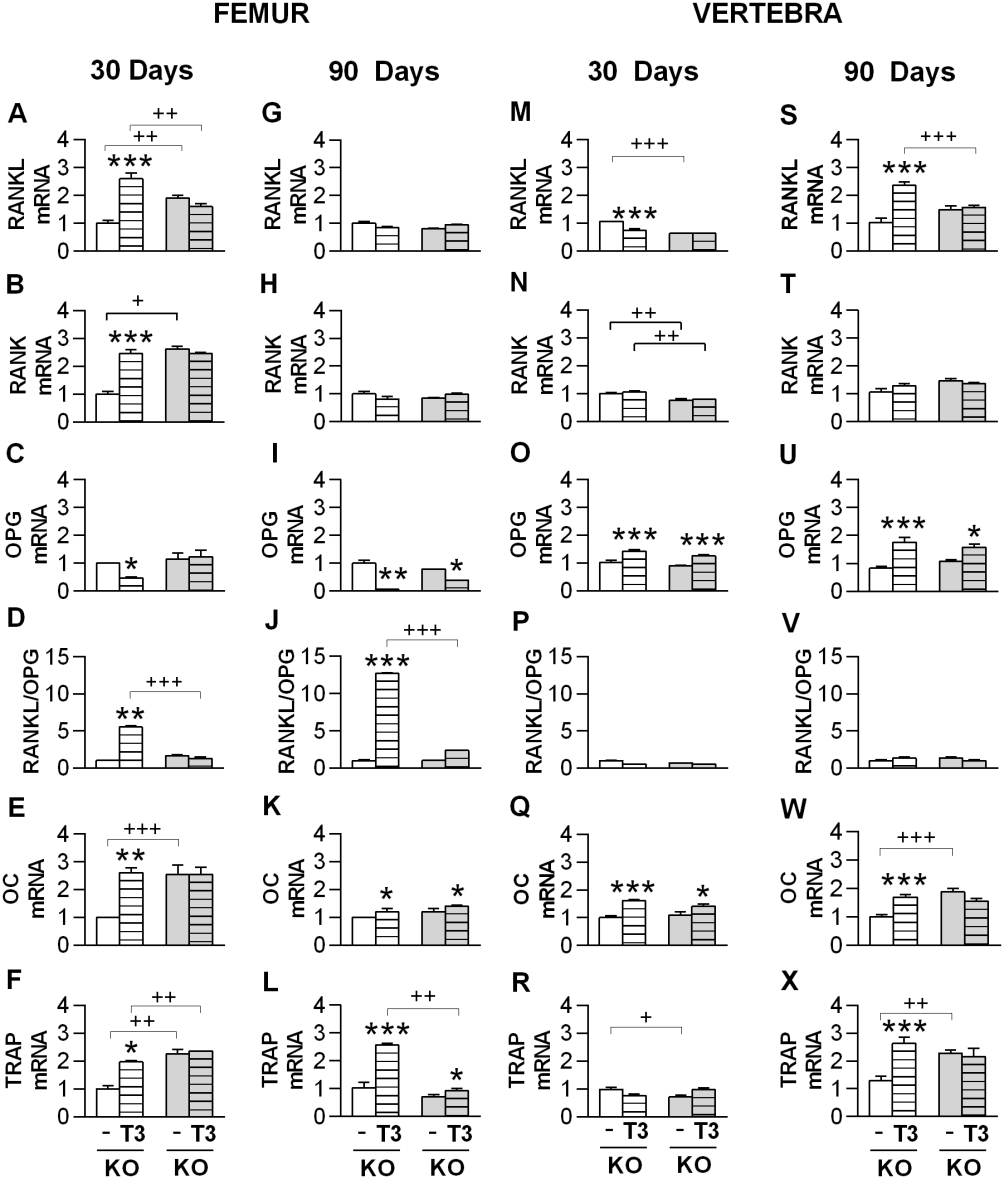
Effect of T3-treatment on the relative mRNA expression of bone metabolism-related genes. (A-L) Genes expressed in the femur. (M-X) Genes expressed in the vertebra. (A-F and M-R) Effect of 30-day treatment. (G-L and S-X) Effect of 90-day treatment. Receptor activator of nuclear factor-ҡB (RANK), RANK ligand (RANKL), osteoprotegerin (OPG), osteocalcin (OC) and tartrate-resistant acid phosphatase (TRAP). mRNA expression was determined by real-time PCR analysis. Animals were treated with saline or a supraphysiological dose of T3 (7 μg·100 g body wt^-1^·day^-1^) for 30 or 90 days. Values are expressed as means ± EPM (*n* = 4 to 5/group). Significance between groups was determined by two-way ANOVA followed by Tukey’s test. *P < 0.05 and **P< 0.01, ***P<0.001 *vs*. the respective saline-treated animals (WT vs. WT+T3, KO vs. KO+T3), ^+^*P*< 0,05, ^++^*P*< 0,01 and ^+++^*P*< 0,001 for differences between WT and KO mice, as indicated.

To further investigate the involvement of α_2C_-AR in bone physiology and in the TH-SNS interaction to control bone remodeling, we evaluate the effect of T3 on the gene expression of the RANKL-RANK-OPG system. RANKL mRNA levels were higher in the femur (95%) and lower in the vertebra (56%) of 30-day saline-treated α_2C_-AR^-/-^mice (60-day old mice), as compared with WT controls (Fig 7A and 7M). These mRNA differences were not observed in 90-day saline-treated animals (120-day-old mice). We also observed that 30 days of T3 treatment increased RANK and RANKL mRNA expression (2.5- and 2.6-fold) and decreased OPG mRNA (50%) expression in the femur of WT mice, but not in the femur of KO mice (Fig 7A–7C). These effects were not observed when the T3 treatment was increased to 90 days (Fig 7G–7I). The single effect of this longer treatment was the decrease of OPG mRNA expression (Fig 7I), which was observed in the femur of both WT and KO animals (93% and 50%, respectively). The RANKL/OPG ratio was drastically increased by 30 and 90 days of T3 treatment (4 and 12 times, respectively) in the femur of WT but not KO animals (Fig 7D and 7J). The responses of the RANKL-RANK-OPG system to T3 treatment were quite different in the vertebra. RANKL was decreased in 40% by 30 days of T3 treatment and increased in 2.3-fold by 90 days of T3 treatment in WT animals, which was not observed in KO animals (Fig 7M and 7S). RANK and RANKL/OPG ratio were not affected by any treatment (Fig 7N, 7T, 7P and 7V), whereas OPG mRNA expression was increased (from 42% to 100%) by 30 and 90 days of T3 treatment both in WT and KO mice (Fig 7O and 7U). We also evaluated the mRNA expression of the bone formation- and bone resorption-related genes, osteocalcin (OC) and tartrate-resistant acid phosphatase (TRAP), respectively. T3 treatment, 30- and 90-day long, increased OC mRNA expression both in the femur (2.6- and 1.2-fold, respectively) and vertebra (1.6- and 1.7-fold, respectively) of WT mice (Fig 7E, 7K, 7Q and 7W); this effect of T3 was not observed in the femur of α_2C_-AR^-/-^mice after 30 days of treatment (Fig 7E) and in the vertebra of KO mice after 90 days of treatment (Fig 7W), revealing certain KO resistance to the T3-induction of OC mRNA expression, a well known effect of T3 [13]. TRAP mRNA expression was significantly increased (2- to 2.5-fold) by 30 and 90 days of T3-treatment in the femur and by 90 days of T3-treatment in the vertebra of WT animals. These effects were cleared impaired by the lack of α_2C_-AR (Fig 7F, 7L and 7X).

## Discussion

We recently showed that young adult mice with global double gene inactivation of α_2A_-AR and α_2C_-AR (α_2A/2C_-AR^-/-^) present a phenotype of HBM [27] and are resistant to the osteopenic effects of thyrotoxicosis [39]. These findings strongly suggest that α_2A_-AR and/or α_2C_-AR signaling mediate actions of the SNS in the skeleton and that TH, when in toxic levels, interact with this system to cause bone loss. To gain insights regarding the specific roles of the different α_2_-AR subtypes in these processes, we evaluated, in the present study, the bone phenotype of mice with the global single gene inactivation of α_2C_-AR subtype (α_2C_-AR^-/-^ mice), and the skeletal responses of these KO animals to chronic conditions of TH excess (30 or 90 days of thyrotoxicosis). Similarly to α_2A/2C_-AR^-/-^ mice, α_2C_-AR^-/-^ animals also present sympathetic over activity with increased circulating and urine levels of epinephrine [47].

Interestingly, the bone phenotype of α_2C_-AR^-/-^ mice showed to be different from that of α_2A/2C_-AR^-/-^ mice. While the latter present a generalized phenotype of HBM [27], α_2C_-AR^-/-^ mice present lower trabecular bone mass in the femur compared with WT mice, which was accompanied by decreased bone strength in the femur and tibia. The opposite was observed in the vertebra, where α_2C_-AR^-/-^ mice show increased trabecular bone mass compared with WT animals, likewise α_2A/2C_-AR^-/-^ mice. This heterogeneous bone phenotype presented by α_2C_-AR^-/-^ mice reinforces the hypothesis that the SNS regulates bone remodeling and structure, via α_2C_-AR signaling, but also implies that this regulation occurs in a skeletal site-dependent way. It is important to consider that the lower bone mass observed in the femur of α_2C_-AR^-/-^ mice may be the result of local activation of β_2_-AR, since these animals present a hyperadrenergic state [47]. An intriguing question is why it does not happen in the vertebra of α_2C_-AR^-/-^ mice and in the whole skeleton α_2A/2C_-AR^-/-^ mice. The fact that the double KO animals (α_2A/2C_-AR^-/-^) present a generalized phenotype of HBM, regardless of the elevated sympathetic tone, suggests that α_2A_-AR and/or α_2C_-AR also mediate osteopenic actions of the SNS. Therefore, the lack of both receptors could result in anabolic effects that could overcome the negative effects of β_2_-AR activation in the bone [27]. Following this reasoning, the comparison of α_2C_-AR^-/-^and α_2A/2C_-AR^-/-^mice phenotypes suggests that the lack of only α_2C_-AR is sufficient to surpass the osteopenic effects of β_2_-AR activation in the vertebra, while both α_2A_-AR and α_2C_-AR absences, or the single α_2A_-AR absence, may be necessary to overcome the osteopenic effects of β_2_-AR activation in the femur.

Similarly to α_2C_-AR^-/-^ mice, leptin deficient mice (ob/ob mice) also exhibit a heterogenic skeletal phenotype, with higher cancellous bone mass in lumbar vertebra and lower cancellous bone mass in distal femur metaphysis when compared to WT animals [48]. A possible explanation to the complex skeletal phenotype of ob/ob animals maybe a balance between anabolic actions of peripheral leptin and antiosteogenic actions of hypothalamic leptin [48,49].

The mechanisms of α_2_-AR action in the bone are still unknown. The presence of α_2_ adrenoceptor subtypes in mouse and human bone cells suggest direct actions of the SNS, via α_2_-AR signaling, in the skeleton. This hypothesis is supported by the responsiveness of in vitro mouse calvaria-derived osteoblasts and mouse bone marrow-derived osteoclasts to the selective α_2_–AR agonist clonidine and to the nonspecific α-AR antagonist phentolamine [27,39]. However, given the broad distribution of α_2_ receptors in non-neuronal cells, and specially in the peripheral and CNS (as autoreceptors and heteroceptors), it is much more likely that α_2_-AR mechanisms of action involve both central and local pathways, which are important points to be investigated in future studies.

Further evidence for the involvement of α_2_-AR signaling in the neuro-endocrine regulation of bone remodeling was shown in humans. Mkalar et al, 2010 identified expression of α_2A_-AR in osteoblasts and lining cells and showed associations of the α_2A_-AR gene locus with bone mineral density and with important bone remodeling markers, such as serum C-terminal cross-linking telopeptide of type I collagen,serum cathepsin K and plasma osteocalcin. In addition, α_2A_-AR was shown to be upregulated in osteoblasts derived from osteoporotic patients relative to osteoblasts derived from non-osteoporotic patients [50].

In spite of the heterogeneous bone phenotype, low bone mass in the femur and high bone mass in the vertebra, α_2C_-AR^-/-^ mice presented resistance to the thyrotoxicosis-induced bone deterioration in both skeletal sites, similarly to α_2A/2C_-AR^-/-^ mice [39]. As expected, thyrotoxicosis negatively regulated most of the micro architectural features of the trabecular bone of the distal metaphysis of the femur and vertebral body of L5 in WT animals, what was not observed or occurred in a much lower magnitude in α_2C_-AR^-/-^ mice.^.^ This resistance was very evident in the vertebra of KO animals, where no negative effects of toxic levels of T3 were detected in any μCT parameter, after 30 or 90 days of T3-treatment. On the other hand, two trabecular parameters of the femur (BV/TV and Tb.N), which is a skeletal site known to be very sensitive to TH [45,46], were negatively affected by thyrotoxicosis also in KO animals. However, these osteopenic effects were detected only after 90-days of T3-treatment (whereas they were detected after 30 and 90 days of T3-treatment in WT animals) and in a much lower magnitude than that observed in WT animals, also revealing a reasonable degree of resistance to the thyrotoxicosis-induced osteopenia. In addition, the chronic T3 treatments (30- and 90-day long) also had negative effects on the biomechanical properties (maximum load and ultimate load) of the femur and tibia of WT, but not of KO mice. These findings support a TH-SNS interaction to control bone remodeling and structure. More importantly, these new findings strongly suggest that the mechanism of action of TH to promote bone loss depends on α_2C_-AR subtype signaling. Once again, it is still unknown if the TH-SNS interaction occurs locally (in the skeleton) or at the CNS level. Previous in vitro studies with calvaria-derived osteoblasts, showed that the known negative effects of T3 on cell growth [16,51] were completely absent or reversed in α_2A/2C_-AR^-/-^ cells. Additionally, the combination of T3 with clonidine had an additive effect on the inhibition of WT cell growth, whereas T3 attenuated the positive effect of clonidine on α_2A/2C_-AR^-/-^ cell growth. Altogether, these findings suggest that a TH-SNS interaction involving α_2_-AR signaling may occur in osteoblasts to locally regulate bone remodeling [39].

To further investigate the basis of the complex bone phenotype of α_2C_-AR^-/-^ mice and their resistance to the T3-induced bone weakening, we evaluated the effect of T3 on gene expression of the RANKL-RANK-OPG system, which plays an essential role in the differentiation and activity of osteoclasts [52]. RANKL is expressed by the osteoblasts and is the ligand of RANK, an osteoclast plasma membrane receptor. The RANKL-RANK interaction induces osteoclast formation, function, and survival [52]. OPG is also expressed by the osteoblasts, and is the natural inhibitor of osteoclastic activity, since it binds RANKL and thereby impairs RANKL/RANK association. An interesting result was that RANKL mRNA levels were higher in the femur and lower in the vertebra of 60-day old α_2C_-AR^-/-^mice, as compared with WT controls. This substantiates the lower and higher trabecular bone volume in the femur and vertebra of KO mice, respectively. These mRNA differences, however, were not observed in 120-day-old mice. We also observed that 30 days of T3 treatment increased RANK and RANKL mRNA expression and decreased OPG mRNA expression in the femur of WT mice, but not in the femur of KO mice. These effects, however, were no longer observed when T3 treatment was increased to 90 days. More interestingly, the RANKL/OPG ratio was significantly increased by both 30 and 90 days of T3 treatment in the femur of WT but not KO animals. The responses of the RANKL-RANK-OPG system to T3 treatment were quite different in the vertebra and were fairly similar between WT and KO mice. Altogether, these findings suggest that the RANKL-RANK-OPG system may partially mediate the effects of T3-treatment only in the femur and that this system may need an intact α_2C_-AR signaling to work properly. We also evaluated the mRNA expression of bone turnover-related genes, such as osteocalcin (OC), which is expressed by mature osteoblasts [53], and tartrate-resistant acid phosphatase (TRAP), which is expressed by mature osteoclasts and is involved in bone resorption [54]. As expected, the supraphysiological T3 treatment increased mRNA expression of OC in the femur and vertebra of WT mice, which is a known effect of TH [13]. This effect, however, was much less evident in α_2C_-AR^-/-^mice. TRAP mRNA expression was also significantly increased by T3-treatment both in the femur and vertebra of WT animals, and this effect was cleared impaired by the lack of α_2C_-AR as well. The increases in OC and TRAP mRNA expression reflect an increase in bone turnover. It is well known that TH excess increases both bone formation and resorption, but the latter is favored leading to bone loss [8,55]. The unresponsiveness of OC and TRAP mRNA expression to T3 treatment in KO mice suggests that the lack of α_2C_-AR considerably impairs the thyrotoxicosis-induced activation of both osteoblasts and osteoclasts and, once more, support a TH-SNS interaction to control bone remodeling in a α_2C_-AR-dependent manner.

In summary, we have shown that the global gene inactivation of α_2C_-AR subtype in young adult mice results in a heterogeneous bone phenotype, with decreased bone mass in the femur and increased bone mass in the vertebra. In spite of these differences, the global absence of α_2C_-AR subtype also results in resistance to the deleterious effects of thyrotoxicosis on bone structure and strength of both skeletal sites. Altogether, these results bring new evidence that (i) α_2_-AR signaling mediates sympathetic actions in the skeleton; that (ii) these actions might be dependent on the skeleton site; and that (iii) α_2C_-AR signaling, but not α_2A_-AR signaling, is relevant to the thyrotoxicosis-induced osteopenia. It is important to consider, however, that the KO mice of the present study lack global α_2C_-AR isoform since conception, which may promote indirect developmental defects or adaptations that could explain, at least partially, the bone phenotypes of these mice and the differential responses to thyrotoxicosis. Nevertheless, the findings of the present study show that the SNS actions in the skeleton are much more complex than initially proposed.

## Supporting Information

S1 Fig Effect of T3-treatment on the structural parameters of the cortical bone of the vertebral body of L5 in WT and α_2C_-AR^-/-^ mice assessed by μCT. (A–D) Effect of 30 days of T3 treatment. (E-H) Effect of 90 days of T3 treatment. Animals were treated with saline or a supraphysiological dose of T3 (7 μg 100 g body wt^-1^ day^-1^). Significance between groups was determined by two-way ANOVA followed by Tukey’s test. Values are expressed as means ± SEM (*n* = 10–12 per group). *P <0.05 *vs*. the respective saline-treated animals (WT *vs*. WT+T3, KO *vs*. KO+T3). ^++^*P*< 0.01 for differences between WT and KO mice, as indicated. T.Ar, tissue area; B.Ar, bone area; Ma.Ar, medullary area; Ec.Pm, endocortical perimeter.
(TIF)
